# Evidence of Insulin Resistance in Adult Uncomplicated Malaria: Result of a Two-Year Prospective Study

**DOI:** 10.1155/2014/136148

**Published:** 2014-12-23

**Authors:** Samuel Acquah, Johnson Nyarko Boampong, Benjamin Ackon Eghan Jnr, Magdalena Eriksson

**Affiliations:** ^1^Department of Medical Biochemistry, School of Medical Sciences, University of Cape Coast, Cape Coast, Ghana; ^2^Department of Biomedical and Forensic Sciences, School of Biological Sciences, University of Cape Coast, Cape Coast, Ghana; ^3^Department of Medicine, School of Medical Sciences, Kwame Nkrumah University of Science and Technology, Kumasi, Ghana; ^4^African Institute for Mathematical Science Ghana (AIMS-Ghana), P.O. Box LG 197, Legon-Accra, Ghana

## Abstract

The study aimed at investigating the effects of adult uncomplicated malaria on insulin resistance. Fasting levels of blood glucose (FBG), glycosylated hemoglobin (HbA1c), and serum insulin were measured in 100 diabetics and 100 age-matched controls before and during *Plasmodium falciparum* malaria. Insulin resistance and beta cell function were computed by homeostatic models assessment of insulin resistance (HOMAIR) and beta cell function (HOMAB) formulae, respectively. Body mass index (BMI) was computed. At baseline, diabetics had significantly (*P* < 0.05) higher levels of BMI, FBG, HbA1c, and HOMAIR but lower level of HOMAB than controls. Baseline insulin levels were comparable (*P* > 0.05) between the two study groups. During malaria, diabetics maintained significantly (*P* < 0.05) higher levels of BMI, FBG, and HbA1c but lower levels of insulin and HOMAB than controls. Malaria-induced HOMAIR levels were comparable (*P* > 0.05) between the two study groups but higher than baseline levels. Apart from BMI and HOMAB, mean levels of all the remaining parameters increased in malaria-infected controls. In malaria-infected diabetics, significant (*P* < 0.05) increase was only observed for insulin and HOMAIR but not the other measured parameters. Uncomplicated malaria increased insulin resistance in diabetics and controls independent of BMI. This finding may have implications for the evolution of T2DM in malaria-endemic regions.

## 1. Introduction

Malaria and type 2 diabetes mellitus (T2DM) continue to affect millions of people globally. Whereas T2DM can be considered a truly global phenomenon, malaria seems to be concentrated in relatively less developed economies of the globe. This implies that the economically less developed continents of the globe have to battle with the negative impact of malaria and T2DM separately or synergistically on the health of the populace.

Malaria is an intricate disease viewed as the most detrimental and pervasive parasitic infection worldwide [1]. It impinges on the health and wellbeing of more than 500 million people and results in 1-2 million deaths yearly [1, 2]. According to the current estimates of the World Health Organization (WHO), 219 million cases of the disease occurred in 2010, with associated 660 000 deaths worldwide [3], suggesting a global decline in malaria-related morbidity and mortality. In spite of the decline, the contribution of Africa to the global malaria burden remains unchanged at 80% and 90%, respectively, for malaria-related morbidity and mortality.

Ironically, current report by the International Diabetes Federation (IDF) indicates that the incidence of diabetes is increasing globally [4]. It is projected that, by the year 2030, an estimated 366 million people affected by the disease in 2011 will reach 552 million with 80% of them living in low and middle income countries [4]. These statistics indicate that, in developing countries, the probability of coexistence of diabetes and malaria in the same individual is quite high making the need for studying the interaction between the two health conditions long overdue. Indeed, Danquah et al. [5] established that diabetics have increased risk of getting malaria compared to their nondiabetic controls. With Ghana being a sub-Saharan African country experiencing increased incidence of diabetes, especially, T2DM, while maintaining her endemic status with respect to malaria infection, the interaction between malaria and diabetes in the same individual cannot be overlooked and this may pose additional health challenge that ought to be investigated.

Insulin resistance, which signifies reduced sensitivity of insulin-sensitive cells to insulin-mediated actions, has long been recognized as a key factor in the pathogenesis of T2DM [6]. In a prospective Italian study, Bonora et al. [7] reported insulin resistance as an independent risk factor for cardiovascular disease (CVD) in diabetics. Since then, a number of subsequent studies [8, 9] have linked insulin resistance to various CVD or CVD risk factors.

Apart from a study in Sudan that investigated insulin resistance in the context of childhood malaria [10], all other investigations looked at insulin resistance in terms of lipid accumulation particularly in the development of T2DM.

To the best of our knowledge, no study has considered the effect of malaria on insulin resistance in adult diabetics and nondiabetic controls. In view of the predicted increased incidence of T2DM in sub-Saharan Africa, a continent that is still grappling with malaria-imposed health challenges, coupled with a recent finding that diabetics are more prone to getting malaria [5], we sought to investigate whether uncomplicated malaria in adult could alter insulin resistance and beta cell function in diabetics receiving treatment at the Cape Coast Teaching Hospital (CCTH) compared with age-matched nondiabetic controls.

## 2. Materials and Methods

### 2.1. Study Area

The study was carried out at the Diabetic Clinic of CCTH in the Cape Coast Metropolis. CCTH serves as the referral hospital for the various health facilities in the region. It has a well-structured and active Diabetic Clinic with patients from various parts of the region. The Central Region shares boundaries with Ashanti and Eastern regions to the north, Western region to the west, Greater Accra region to the east, and the Atlantic Ocean to the south. It covers an area of 9,826 km^2^ with Cape Coast as its capital [11]. The region is considered the educational hub of Ghana as the majority of the country's top senior high schools are located in the region. According to the 2010 Population and Housing Census, the regional population stood at 2,201,863 with that of the Cape Coast metropolis being 169,894 [12]. Ecologically, the region is divided into the forest and coastal zones. In terms of climate, Central region is mainly tropical but experiences South West Monsoon and North East Trade winds with average temperature of 21–31°C and annual rainfall figure of 750–1000 mm [11]. The people are mainly farmers and fishermen in the informal sector with a relatively small proportion of the working population in the formal sector [11].

### 2.2. Study Participants and Laboratory Determinations

The study was an outpatient-based comparative type that followed type 2 diabetics and nondiabetic controls over a two-year period for malaria infection. The inclusion criteria used in selecting study participants were being 40 years or older, nonalcoholic, nonsmoker, not suffering from any cardiovascular disease, hepatitis B, C, and HIV, not pregnant, not nursing a baby, with no evidence of hepatic dysfunction, with absence of severe arthritis, lupus, or inflammatory bowel disease, and not undergoing hormonal therapy or having any medical condition that can significantly influence the levels of any of the biomarkers measured.

Diabetics were randomly selected from a database of registered diabetes patients attending the diabetic clinic at CCTH and invited to participate in the study. Controls from the general inhabitants of the Cape Coast metropolis were age-matched with the diabetic group. In all, 200 respondents consisting of 100 diabetics and 100 nondiabetic controls were enrolled for the study in 2011. Before and during malaria infection, 10 mL of venous blood sample was obtained after overnight fast. Blood sample was separated into plasma and serum for determination of various biochemical indices. Whereas plasma samples were analyzed the same day, serum samples were aliquoted appropriately and stored at −80°C till further analysis. Weight and height were measured for computation of body mass index (BMI) using standard protocol.

Fasting blood glucose (plasma) and glycosylated hemoglobin were determined the same day by the BT3000 autoanalyzer with reagents from JAS diagnostics (JAS Diagnostics Inc., USA). Blood glucose determination followed the enzymatic protocol with that of glycosylated hemoglobin relying on the agglutination procedure.

Serum insulin was measured by a commercially available quantitative enzyme-linked immunosorbent assay (ELISA) kit from Calbiotech (Calbiotech Inc., USA) with reagents from the same manufacturer. The specific ELISA is the direct sandwich type. Briefly, samples, standards, and reagents were brought to room temperature. Exactly 25 *μ*L each of the various concentrations of insulin standards (6.25, 12.5, 25, 50, and 100 *μ*IU/mL) was placed in appropriate wells. The same volume of sample was also placed in each well, followed by the addition of 100 *μ*L of 1X enzyme conjugate prepared by diluting 500 *μ*L of 20X enzyme conjugate concentrate with 9500 *μ*L of assay diluent. After thorough mixing, the setup was then incubated for 1 hour. Using an automated microplate washer (Thermo Electron Co-Operation, Finland), the microplate wells were washed 6 times each with 300 *μ*L of 1X wash buffer prepared by mixing 25 mL of 20X wash buffer concentrate with 475 mL of distilled water, after the incubation period. Exactly 100 *μ*L of TMB substrate was then added to each well followed by incubation at room temperature for 15 minutes and addition of 50 *μ*L stop solution. Absorbance was then read immediately at 450 nm using a Multiscan Microplate Reader (Thermo Scientific, Finland). Appropriate standard curve was prepared from absorbance values of the insulin standards. The concentration of insulin in sample was subsequently determined from the standard curve.

HOMAIR and HOMAB were estimated from glucose and insulin levels by the formulae of Matthews et al. [13] as HOMAIR = fasting insulin (*μ*U) × fasting glucose (mmol/L)/22.5:

(1) $$\begin{array}{c} \text{HOMAB}=\frac{20\times \text{fasting}\;\;\text{insulin}\;\left(\mu \text{U}/\text{mL}\right)}{\text{fasting}\;\;\text{glucose}\;\left(\text{mmol}/\text{L}\right)-3.5}. \end{array}$$

Malaria was diagnosed by the CareStart Malaria HRP2Pf rapid diagnostic test kit manufactured by Access Bio (Access Bio Inc., USA). The CareStart Malaria HRP2Pf has been found to exhibit high sensitivity and specificity and correlates strongly with microscopy [14–16] in diagnosing* falciparum* malaria.

Diabetics were already diagnosed type 2 diabetic patients receiving appropriate treatment at CCTH excluding exogenous insulin.

### 2.3. Statistical Analysis

Data obtained were analyzed by SPSS software version 17. Data are presented as mean ± standard deviation except the HOMAIR values that were log-transformed and presented as geometric mean ± standard deviation. Mean levels of measured parameters were compared between study groups and between genders with independent sample *t*-test. Mean levels of parameters before and during malaria were compared across groups by one-way ANOVA with Tukey post hoc test. Pearson correlation test was used to assess linear relationship between any pair of measured parameters. A stepwise linear regression model was then applied to identify independent predictors of malaria-induced HOMAIR. A *P* value < 0.05 was considered statistically significant.

## 3. Results

Of the 200 respondents, 100 (70 diabetics and 30 controls) had malaria after a two-year follow-up. Age and baseline serum insulin level did not differ (*P* > 0.05) between the defined study groups (Table 1). However, diabetics had significantly (<0.001) higher levels of FBG, HbA1_c_, HOMAIR, and BMI but lower level of HOMAB than their nondiabetic counterpart. Stratification of baseline data by intragroup gender revealed a comparable (*P* > 0.05) mean level of all the measured parameters between the sexes in both study groups except FBG and BMI. FBG was higher (*P* = 0.023) in control males compared with their female counterpart with BMI being higher (*P* < 0.001) in diabetics females in comparison with their male counterpart (data not shown).

**Figure 1:**
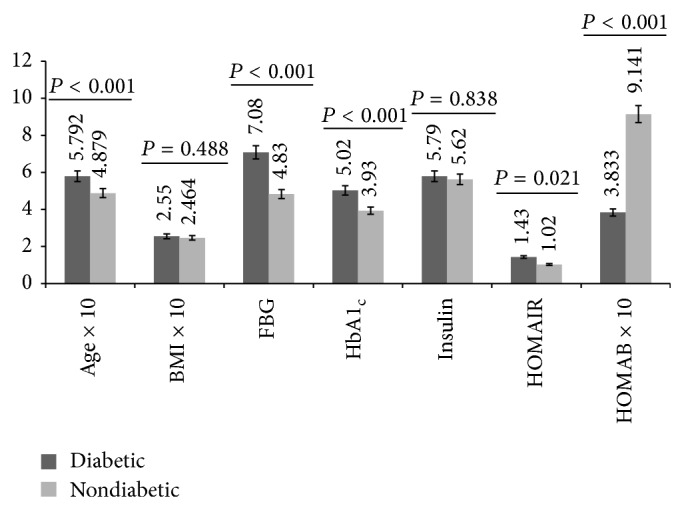
Age, BMI, glycemic indicators, insulin, HOMAIR, and HOMAB levels of male respondents by study group.

**Figure 2:**
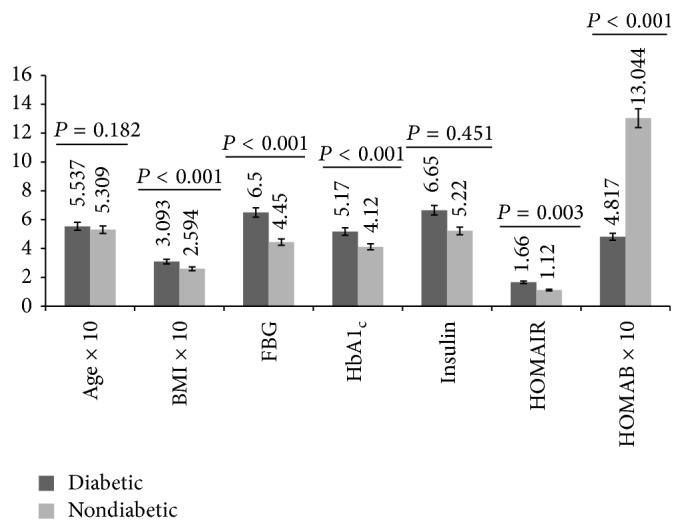
Age, BMI, glycemic indicators, insulin, HOMAIR, and HOMAB levels of female respondents by study group.

Intergroup gender comparison of baseline data showed that diabetic males were significantly (*P* < 0.05) older and had higher levels of FBG, HbA1_c_, and HOMAIR but lower level of HOMAB than control males (Figure 1). With respect to the females, a similar trend was observed with exception of age and BMI which were comparable (*P* = 0.182) and higher (*P* < 0.001), respectively, in diabetic respondents (Figure 2). Baseline insulin level did not (*P* > 0.05) show either intergroup or intragroup gender variation (Figures 1 and 2).

In the presence of malaria, diabetics maintained significantly (*P* < 0.001) higher mean levels of BMI, FBG, and HbA1_c_ but lower levels of serum insulin and HOMAB than controls (Table 2). Interestingly, the two groups did not differ (*P* = 0.563) in their malaria-induced HOMAIR levels (Table 2).

Analysis of variance comparison of means revealed that mean levels of all the measured parameters differed significantly (*P* < 0.001) across groups with and without malaria (Table 3).

Tukey's post hoc HSD test rather showed that, apart from the mean HOMAIR and serum insulin that increased significantly (*P* < 0.05) in diabetic respondents during malaria, the mean levels of all the other measured parameters were comparable (*P* > 0.05) before and during malaria (Table 4). However, in the control group, significantly (*P* < 0.05) higher mean levels were observed for all the measured parameters except BMI and HOMAB (Table 4).

Malaria-induced HOMAIR correlated positively and moderately (*R* = 0.435; *P* = 0.001) but strongly (*R* = 0.901; *P* < 0.001) with malaria-induced HOMAB and insulin, respectively, in the diabetic group. In a subsequent stepwise linear regression model, malaria-induced HOMAB and insulin were significant (*P* < 0.05; *R* ^2^ = 0.954; adjusted *R* ^2^ = 0.915) independent predictors of HOMAIR, jointly explaining over 91% of the observed variation.

Similar analyses in the control group showed positive correlation of malaria-induced HOMAIR level with malaria-induced HOMAB (*R* = 0.479; *P* = 0.013), malaria-induced insulin (*R* = 0.978; *P* < 0.001), and baseline insulin level (*R* = 0.634; *P* = 0.011). Also, malaria-induced HOMAB correlated positively with baseline HOMAB (*R* = 0.66; *P* = 0.002) and malaria-induced insulin level (*R* = 0.686; *P* < 0.001). Above all, baseline HOMAB correlated positively with malaria-induced insulin level (*R* = 0.498; *P* = 0.025). As expected, a stepwise linear regression model revealed that malaria-induced HOMAB, malaria-induced insulin, and baseline insulin levels were independent (*R* ^2^ = 0.996; adjusted *R* ^2^ = 0.994; *P* < 0.001) predictors of the observed HOMAIR level during malaria infection. The model explained over 99% of the observed variation in HOMAIR levels in controls who had malaria.

Conspicuously, no significant (*P* > 0.05) association was observed between BMI and any of the measured indices of insulin sensitivity or glycemia.

## 4. Discussion

This study investigated malaria-induced insulin resistance in adult diabetics and controls in the Cape Coast metropolis of the Central region of Ghana. The comparable mean age of respondents between the two study groups in the current study appears contrary to a study in Kumasi that found higher age for diabetics than nondiabetic controls [5] though the higher age of diabetic males compared with their control counterpart in the present study supports the Kumasi study [5]. This seeming age-specific contradictory observation in the current study compared to that of Danquah et al. [5] could be ascribed to differences in sample size, study design, and characteristics of respondents in the two studies. Whereas the current study was limited to 200 adults aged 40 years and above, that of Danquah et al. [5] included a much larger number of adults 18 years and beyond. Indeed, gender-specific consensus on age at diagnosis as a substitute for age of onset of T2DM is yet to be reached by the scientific community. Kolb et al. [18] observed lower age of onset for men contrary to earlier one [19] that reported rather higher age for men. Recently, a number of reports [20, 21] have found comparable age of onset for males and female diabetics in line with the current study. The varied conclusions in literature regarding age of onset of T2DM between the sexes could be attributed to possible differences in racial, genetic, and environmental factors related to the evolution of the disease in various populations [22–24].

As expected, diabetics in the current study recorded higher baseline mean levels of FBG and HbA_1c_ than controls except that the observed mean levels for diabetic respondents were lower than those reported by other researchers in China [21], India [20], Nigeria [25], and United States of America [26]. The rather low levels of baseline glycemic indicators in the current study as opposed to earlier reports [20, 21, 25] could be due to differences in characteristics of enrolled respondents with respect to duration of disease, adherence to prescribed treatment regimen, and analytical method employed in determination of the glycemic parameters. In the presence of malaria, levels of the glycemic indicators in both study groups increased with diabetics maintaining their higher levels compared with controls as observed at baseline. However, the extent of increase due to malaria was only appreciable in the nondiabetic group suggesting that diabetics in the current study did not respond to malaria by increasing blood glucose level. The appreciable increase in FBG due to malaria in the control arm of the current work supports earlier finding in Netherlands [27] and has been attributed to increased gluconeogenesis. Considering the fact that HbA1_c_ is a marker for relative long-term blood glucose control, its appreciable increase due to* Plasmodium falciparum* malaria in the control group of the current study is an indication that the increased FBG was not transient. Interestingly, the comparable levels of FBG and HbA1_c_ of diabetics before and during malaria could be due to treatment effect since most diabetics were on metformin, an antidiabetic drug with acknowledged suppression effect on hepatic gluconeogenesis [28, 29]. On the other hand, the slight increase could be a pointer to a probable attenuated inhibition of gluconeogenesis by metformin, suggesting that increased intensity of infection may eliminate the metformin-induced inhibition of hepatic gluconeogenesis and thus result in significant increase in FBG and HbA1_c_. This view requires further studies to be proven in the light of a recent finding that associates HbA1_c_ with intima-media thickness, a surrogate marker for CVD risk [30], and a larger study [31] suggesting that every 1% rise in HbA1_c_ is associated with 1.67 risk of developing coronary heart disease in apparently healthy adults. Thus, with diabetics being already at risk for CVD, further investigations ought to be carried out to ascertain the clinical relevance of the slightly elevated FBG and HbA1_c_ levels as a result of malaria found in the current study.

Insulin resistance, defined as the reduced response of insulin sensitive cells to the action of insulin, has been amply demonstrated to be central to the development of T2DM [32] and CVD [33]. The condition which coexists with reduced beta cell secretory function has been observed in the Ghanaian population [34]. It is ideally determined by the hyperinsulinemic-euglycemic clamp technique developed by DeFronzo, Tobin, and Andres over three decades ago [35]. However, this technique, which directly measures insulin resistance and is widely accepted as the gold standard, is time and labor intensive and costly and requires advanced technical expertise [36], discouraging its use in a routine manner in clinical practice and research with insulin resistance as secondary objective. To this end, a number of indirect mathematical models have been developed with the HOMAIR and HOMAB models, developed by Matthews and colleagues [13] gaining broad acceptance by the scientific community. The model, which depends on blood levels of insulin and glucose, has good correlation with the gold standard technique [37]. Although the use of HOMAIR-dependent diagnosis of insulin resistance in different populations varies with cutoffs [38–40], a value of 2.6 appears generally acceptable as the threshold above which insulin resistance is diagnosed [41]. Generally, HOMAIR values in diabetics have been reported to be higher than nondiabetic controls [38, 38, 40] as observed at baseline in the current study. However, malaria-induced increased mean HOMAIR values above 3.2 in diabetics and controls observed in the current work have never been reported in the adult populations to the best of our knowledge. Only one study in Sudan [10] reported insulin resistance in children with severe complicated malaria, the form of the disease normally absent in semi-immune adults living in malaria-endemic countries like Ghana. As such, our finding that uncomplicated malaria in semi-immune adults could raise HOMAIR levels by over 120% and 200% in diabetic and nondiabetic controls, respectively, to levels above the accepted 2.6 value is novel and signifies overt insulin resistance due to* falciparum* malaria. This observation, notably, in the nondiabetic group may have implications for the development of T2DM in malaria-endemic countries. With the possibility of multiple episodes of clinical malaria in the same individual, the corresponding insulin resistance per episode could predispose the affected people to the development of overt T2DM with time since insulin resistance precedes development of overt T2DM [32]. This is because the significantly increased mean HOMAIR values found in malaria-infected respondents in the current work did not appear to have resulted in a significantly increased secretory function of their beta cells as determined by HOMAB. Thus, although insulin level increased significantly due to malaria, probably to compensate for the increased resistance, the level was not high enough to overcome the observed resistance. More so, insulin secretion may not be perpetually increased to respond to malaria-induced glucose increase, envisaged in multiple malaria episodes as the beta cells can theoretically be apoptosed by persistent malaria-induced hyperglycemia, a scenario that can result in explicit T2DM in nondiabetic individuals. In the case of diabetics, malaria could attenuate the expected pharmacologic effects of the hypoglycemic drugs, increasing their risk of CVD as their insulin resistance increases, as suggested by the observed HOMAIR in the current report.

Interestingly, the various measures of glycemia and insulin sensitivity did not associate with BMI, suggesting that general fat accumulation played no significant role in the observed insulin resistance in the current study.

Based on the above we propose that malaria-induced insulin resistance may contribute to the predicted higher increased incidence of T2DM for malaria-endemic sub-Saharan African countries [4]. Hence, control measures against malaria may contribute to reduced incidence of T2DM.

## 5. Conclusion

Uncomplicated* falciparum* malaria caused insulin resistance in diabetic and nondiabetic semi-immune adults, independent of BMI. This finding has implications for the evolution of T2DM in malaria-endemic regions of the globe.

## Figures and Tables

**Table 1:** Age, BMI, glycemic indicators, HOMAIR, and HOMAB levels of respondents by study group.

Parameter	Diabetic *N* = 100	Nondiabetic *N* = 100	*P* value
Age (years)	53.43 ± 6.57	52.88 ± 10.29	0.778
BMI (kg/m^2^)	28.50 ± 4.65	26.09 ± 5.34	0.025^*^
FBG (mmol/L)	6.68 ± 1.46	4.54 ± 1.17	<0.001^*^
HbA1_c_ (%)	5.12 ± 1.03	4.07 ± 1.02	<0.001^*^
Insulin (*μ*IU/mL)	5.70 ± 1.69	5.33 ± 1.72	0.838
HOMAIR	1.59 ± 0.19	1.08 ± 0.18	<0.001^*^
HOMAB	43.90 ± 25.31	116.90 ± 21.72	<0.001^*^

Figures represent mean ± standard deviation; BMI: body mass index; FBG: fasting blood glucose; HbA1_c_: glycosylated hemoglobin; HOMAIR: homeostatic model assessment of insulin resistance; HOMAB: homeostatic model assessment of beta cell secretion; ^*^significant *P* value; *N*: number of respondents.

**Table 2:** BMI, FBG, HbA1_c_, insulin, HOMAIR, and HOMAB levels of respondents during malaria by study group.

Parameter	Diabetic *N* = 70	Nondiabetic *N* = 30	*P* value
FBG (mmol/L)	7.12 ± 0.60	5.27 ± 0.60	<0.001^*^
BMI (kg/m^2^)	27.98 ± 4.53	26.01 ± 5.75	0.026^*^
HbA1_c_ (%)	5.67 ± 1.03	4.63 ± 1.07	<0.001^*^
Insulin (*μ*IU/mL)	10.57 ± 5.27	15.45 ± 8.60	0.02^*^
HOMAIR	3.58 ± 0.34	3.32 ± 0.55	0.563
HOMAB	69.74 ± 25.52	189.15 ± 21.40	<0.001^*^

Figures represent mean ± standard deviation; BMI: body mass index; FBG: fasting blood glucose; HbA1_c_: glycosylated hemoglobin; HOMAIR: homeostatic model assessment of insulin resistance; HOMAB: homeostatic model assessment of beta cell secretion; ^*^significant *P* value.

**Table 3:** FBG, HbA1_c_, HOMAIR, and HOMAB levels of respondents with and without malaria.

Parameter	Diabetic		Nondiabetic		*F* value	*P* value
	DM (*N* = 70)	BM (*N* = 100)	DM (*N* = 30)	BM (*N* = 100)		
BMI (kg/m^2^)	27.98 ± 4.53	28.50 ± 4.65	26.01 ± 5.75	26.09 ± 5.34	10.314	0.01^*^
FBG	7.12 ± 0.06	6.68 ± 1.46	5.27 ± 0.06	4.54 ± 1.17	35.564	<0.001^*^
HbA1_c_	5.67 ± 1.03	5.12 ± 1.03	4.63 ± 1.07	4.07 ± 1.02	16.978	<0.001^*^
Insulin	10.57 ± 5.27	5.79 ± 1.78	15.45 ± 8.60	5.33 ± 1.72	11.616	<0.001^*^
HOMAIR	3.58 ± 0.34	1.59 ± 0.19	3.32 ± 0.55	1.08 ± 0.18	8.473	<0.001^*^
HOMAB	69.74 ± 6.09	43.90 ± 25.30	189.15 ± 21.40	116.90 ± 21.70	6.677	<0.001^*^

Figures represent mean ± standard deviation; BMI: body mass index; FBG: fasting blood glucose (mmol/L); HbA1_c_: glycosylated hemoglobin (%); HOMAIR: homeostatic model assessment of insulin resistance; HOMAB: homeostatic model assessment of beta cell secretion; DM: during malaria; BM: before malaria; ^*^significant *P* value; *N*: number of respondents.

**Table 4:** Tukey's HSD comparison of mean levels of parameters in respondents with and without malaria.

Parameter	Malaria	No malaria	*P* value
	Diabetic		
	*N* = 70	*N* = 100	

BMI (kg/m^2^)	27.98 ± 4.53	28.50 ± 4.65	0.74
FBG (mmol/L)	7.12 ± 0.06	6.68 ± 1.46	0.377
HbA1_c_ (%)	5.67 ± 1.03	5.12 ± 1.03	0.139
Insulin (*μ*IU/mL)	10.57 ± 0.73	5.79 ± 1.78	0.011^*^
HOMAIR	3.58 ± 0.34	1.59 ± 0.19	0.009^*^
HOMAB	69.74 ± 6.09	43.90 ± 2.53	0.364

	Nondiabetic		
	*N* = 30	*N* = 100	

BMI (kg/m^2^)	26.01 ± 5.75	26.09 ± 5.34	0.81
FBG (mmol/L)	5.27 ± 0.06	4.54 ± 1.17	0.02^*^
HbA1_c_ (%)	4.63 ± 1.07	4.07 ± 1.02	0.032^*^
Insulin (*μ*IU/mL)	15.45 ± 2.04	5.33 ± 1.72	0.006^*^
HOMAIR	3.32 ± 0.55	1.08 ± 0.18	0.009^*^
HOMAB	189.15 ± 21.40	116.90 ± 21.70	0.169

Figures represent mean ± standard deviation; BMI: body mass index; FBG: fasting blood glucose; HbA1_c_: glycosylated haemoglobin, HOMAIR: homeostatic model assessment of insulin resistance, HOMAB: homeostatic model assessment of beta cell secretion; ^*^significant *P* value; *N*: number of respondents.
